# Genome-wide identification, phylogeny and expressional profiles of mitogen activated protein kinase kinase kinase (MAPKKK) gene family in bread wheat (*Triticum aestivum* L.)

**DOI:** 10.1186/s12864-016-2993-7

**Published:** 2016-08-22

**Authors:** Meng Wang, Hong Yue, Kewei Feng, Pingchuan Deng, Weining Song, Xiaojun Nie

**Affiliations:** 1 State Key Laboratory of Crop Stress Biology in Arid Areas, College of Agronomy and Yangling Branch of China Wheat Improvement Center, Northwest A&F University, 3 Taicheng Road, Yangling, 712100 Shaanxi China; 2 Australia-China Joint Research Centre for Abiotic and Biotic Stress Management in Agriculture, Horticulture and Forestry, Yangling, 712100 Shaanxi China

**Keywords:** Wheat, MAPKKKs, Gene family, Expression profiles

## Abstract

**Background:**

Mitogen-activated protein kinase kinase kinases (MAPKKKs) are the important components of MAPK cascades, which play the crucial role in plant growth and development as well as in response to diverse stresses. Although this family has been systematically studied in many plant species, little is known about MAPKKK genes in wheat (*Triticum aestivum* L.), especially those involved in the regulatory network of stress processes.

**Results:**

In this study, we identified 155 wheat MAPKKK genes through a genome-wide search method based on the latest available wheat genome information, of which 29 belonged to MEKK, 11 to ZIK and 115 to Raf subfamily, respectively. Then, chromosome localization, gene structure and conserved protein motifs and phylogenetic relationship as well as regulatory network of these TaMAPKKKs were systematically investigated and results supported the prediction. Furthermore, a total of 11 homologous groups between A, B and D sub-genome and 24 duplication pairs among them were detected, which contributed to the expansion of wheat MAPKKK gene family. Finally, the expression profiles of these MAPKKKs during development and under different abiotic stresses were investigated using the RNA-seq data. Additionally, 10 tissue-specific and 4 salt-responsive TaMAPKKK genes were selected to validate their expression level through qRT-PCR analysis.

**Conclusions:**

This study for the first time reported the genome organization, evolutionary features and expression profiles of the wheat MAPKKK gene family, which laid the foundation for further functional analysis of wheat MAPKKK genes, and contributed to better understanding the roles and regulatory mechanism of MAPKKKs in wheat.

**Electronic supplementary material:**

The online version of this article (doi:10.1186/s12864-016-2993-7) contains supplementary material, which is available to authorized users.

## Background

Mitogen-activated protein kinase (MAPK) cascades play the crucial role in plant growth and development as well as in response to stresses, which are highly conserved in the signal transduction pathway in eukaryote [1]. The MAPK pathway included three main protein kinase members, namely MAPK kinase kinases (MAPKKK or MEKK), MAPK kinases (MKK or MEK) and MAPKs (MPK). They achieved the function through sequentially being phosphorylated. Upstream signals firstly activated the MAPKKKs, which in turn the MAPKKKs activated the MAPKKs and then specific MAPKs were activated by the MAPKKs. Eventually, the activated MAPKs phosphorylated transcription factors, enzymes or other signaling components to modulate the expression of downstream genes to complete signal amplification [2, 3]. It has been demonstrated that MAPK cascades played a vital role in cell division, growth and differentiation [4, 5], hormone response [6], plant immunity [7, 8], biotic and abiotic stress response and so on [9–11]. To date, extensive studies have been conduct to systematically investigate the MAPKKK gene family in many plant species and it is reported that there were 74 putative MAPKKK genes in maize (*Zea mays*), 75 in rice (*O. sativa*), 78 in cotton (*G. raimondii*) and 80 in Arabidopsis (*A. thalianna*), respectively [12–15].

Wheat is one of the most important crops worldwide, occupying 17 % of cultivated lands and serving as the staple food source for 30 % of the human population all over the world [16, 17]. Genetically, wheat is an allohexaploid species (2n = 6x = 42), which has a complex original and evolutionary history, derived from three diploid donor species through two naturally interspecific hybridization events. The initial hybridization event was occurred between A genome donor (*T. urartu*, AA; 2n = 14) and B geome donor (*Aegilops speltoides*, SS; 2n = 14) to produce the allotetraploid (AABB, *T. turgidum L*) about 0.2 MYa ago, and then the AABB donor crossed with the D genome donor (*A. Tauschii* Coss) to form the allohexaploid wheat (AABBDD) about 9000 years ago [18]. As a result, wheat possesses a large and complex genome with three homologous genomes (A, B and D) and the size more than 17 Gb, which makes it a huge challenge to conduct genomic study in wheat. But, as the newly formed polyploidy, wheat is considered as an ideal model for chromosome interaction and polyploidization studies in plants [19, 20]. Recently, the draft genome sequencing of hexaploid wheat Chinese Spring (CS) was completed using the chromosome-based strategy, which laid the foundation to identify wheat gene family at the genome-level and also to discern the homologous copies in these three sub-genomes [17]. The retention and dispersion of homologous gene will provide the indispensable information about chromosome interaction during polyploidization [21, 22].

At present, no systematical investigation of MAPKKK gene family has been performed in wheat. In light of the functional significance of this family, an in silico genome-wide search was conducted to identify wheat MAPKKK gene family in this study. Then, the chromosome localization, gene structure, conserved protein domain, phylogenetic relationship as well as expression profiles and regulatory network were systematically analyzed in the putative wheat MAPKKK genes to reveal the evolutionary and functional features of these genes. Our study will provide a basis for further functional analysis of the wheat MAPKKK genes, and will contribute to better understanding the molecular mechanism of MAPKKKs involving in regulating growth and development as well as stress processes in wheat.

## Methods

### Identification of MAPKKK gene family in wheat

The wheat MAPKKK gene family was identified following the method as described by Rao et al with some modifications [13]. First, all the wheat protein sequences available were downloaded from the Ensemble database (http://plants.ensembl.org/index.html) to construct a local protein database. Then, this database were searched with 304 known MAPKKK gene sequences collected from *A.thaliana* (80), *O. sativa* (75), *Z. mays* (74) and *B.distachyon* (75) using the local BLASTP program with an e-value of 1e-5 and identity of 50 % as the threshold. Furthermore, all the MAPKKK sequences were aligned and the obtained alignments were used to construct a HMM profile using the hmmbuild tool embedded in HMMER3.0 (http://hmmer.org/download.html), and then the HMM profile were used to search the local protein database using the hmmsearch tool. HMMER and BLAST hits were compared and parsed by manual editing. Furthermore, a self-blast of these sequences was performed to remove the redundancy and the remaining sequences were considered as the putative TaMAPKKK proteins, which then were submitted to the NCBI Batch CD-search database (http://www.ncbi.nlm.nih.gov/Structure/bwrpsb/bwrpsb.cgi) and PFAM databases (http://pfam.xfam.org/) to confirm the presence and integrity of the kinase domain. Finally, all the obtained sequences were verified the existence by BLASTN similarity search against the wheat ESTs deposited in NCBI database. The theoretical pI (isoelectric point) and Mw (molecular weight) of the putative TaMAPKKK were calculated using compute pI/Mw tool online (http://web.expasy.org/compute_pi/). Subcellular localization of each TaMAPKKK cascade kinases were predicted using the TargetP software of the CBS database [23].

### Multiple sequence alignments and phylogenetic analysis

Multiple sequence alignments were generated using ClustalW tool [24]. To investigate the evolutionary relationship among MAPKKK proteins, a neighbor-joining (NJ) tree was constructed by MEGA 6.0 software based on the full-length of MAPKKK protein sequences [25]. Bootstrap test method was adopted and the replicate was set to 1000.

### Gene structure construction, protein domain and motif analysis

The gene structure information were got from Ensemble plants database (http://plants.ensembl.org/index.html) and displayed by Gene Structure Display Server program (GSDS: http:/gsds.cbi.pku.edu.cn/). The protein domains and motifs in the MAPKKKs were predicted using InterProScan against protein databases (http://www.ebi.ac.uk/interpro/). The schematic representing the structure of all members of TaMAPKKKs was based on the InterProScan analysis.

### Chromosomal locations and gene duplication

Genes were mapped on chromosomes by identifying their chromosomal position provided in the wheat genome database. Gene duplication events of MAPKKK genes in wheat were investigated based on the following three criteria: (a) the alignment covered >80 % of the longer gene; (b) the aligned region had an identity >80 %; and (c) only one duplication event was counted for the tightly linked genes [12, 26]. In order to visualize the duplicated regions in the *T. aestivum* genome, lines were drawn between matching genes using Circos-0.67 program (http://circos.ca/).

### Identification of cis-regulatory elements

To investigate the cis-regulatory elements, the upstream regions (2 kbp) of all wheat MAPKKK genes were extracted, which were considered as the proximal promoter regions for the individual wheat MPKKK genes. Then, all the sequences were submitted to PlantCARE database (http://bioinformatics.psb.ugent.be/webtools/Plantcare/html/) to identify the putative cis-acting regulatory elements.

### Network interaction analysis

The interaction network which the TaMAPKKK genes involved were investigated based on the orthologous genes between Wheat and Arabidopsis using the AraNet V2 tool (http//www.inetbio.org/aranet/). Then, enrichment analysis was implemented by BiNGO, a cytoscape plugin, for gene ontology analysis and identifying processes and pathways of specific gene sets. Over-represented GO full categories were identified with a significance threshold of 0.01.

### The MAPKKK gene expression analysis by RNA-seq data

To study the expression of TaMAPKKK genes in different organs and response to stress, transcriptome sequencing data obtained from WHEAT URGI (https://urgi.versailles.inra.fr/files/RNASeqWheat/) and NCBI Sequence Read Archive (SRA) database were used to investigate the differential expression of TaMAPKKKs. The accession numbers and sample information of the used data were listed in Additional file 1. TopHat and Cufflinks were used to analyze the genes’ expression based on the RNA-seq data [27]. The FPKM value (fragments per kilobase of transcript per million fragments mapped) was calculated for each MAPKKK gene, the log10-transformed (FPKM + 1) values of the 155 TaMAPKKK genes were used for heat map generation. And fold change cutoff of two and p-value < 0.05, q-value < 0.05 were taken as statistically significant threshold [28, 29].

### Plant materials, growth conditions, and treatments

The plants of wheat cultivar ‘CS’ were reared in growth chambers at 23 ± 1 °C with a photoperiod of 16 h light/8 h dark. The roots, stems, leaves, spikes (1 d before flowering), and grains (10d after pollination) were collected from flowering plants for tissue expression analysis. One-week-old seedlings which consisted with RNA-seq data were treated by 150 mM NaCl which represented salt treatment, and the seedlings grown under normal condition were used as control. The leaves of seedlings under salt and also control conditions were collected at 0, 6, 12, 24 and 48 h after treatment. All the plant samples from two biological replicates were frozen in liquid nitrogen immediately and stored at −80 °C for RNA isolation.

### RNA isolation and qRT-PCR analysis

The total RNA was extracted using Plant RNA Kit reagent (Omega Bio-Tek, USA) according to the manufacturer’s instructions. The RNA integrity was checked by electrophoresis on 1.0 % agarosegels stained with ethidium bromide (EB). The first strand cDNAs were synthesized using a Vazyme Reverse Transcription System (Beijing, China) following the manufacturer’s protocol. Real-time PCR analyses were performed using the primer pairs listed in Additional file 2. Two biological and three technical replicates for each sample were obtained using the real-time PCR system (BIO-RAD CFX96, USA). The β-actin gene was used as internal reference for all the qRT–PCR analysis. Each treatment was repeated three times independently. The expression profile was calculated from the 2^–△△CT^ value [ΔΔCT = (CT_target/salt_ – CT_actin/salt_) – (CT_target/control_ – CT_actin/control_)] [30].

## Results and discussion

### Genome-wide Identification of MAPKKK Family in Wheat

Availability of the genome sequence made it possible for the first time to identify all the MAPKKK family members in wheat. Using the method as described above, a total of 155 genes with the complete kinase domain were identified as the MAPKKK members in the wheat genome. Since there is no standard nomenclature, the predicted wheat MAPKKK genes were then designated as TaMAPKKK1 to TaMAPKKK155 based on the blast scores. It was notable that wheat possessed the largest MAPKKK gene family among the reported species (Table 1), which may be the result of its allohexaploid genome and complex evolutionary process.

**Table 1:** Comparison of the gene abundance in three subfamilies of MAPKKK genes in different plant species

Species	Raf	MEKK	ZIK	Total
Wheat	115	29	11	155
Arabidopsis	48	21	11	80
Rice	43	22	10	75
Maize	46	22	6	74
Brachypodium	45	24	6	75
Tomato	40	33	16	89
soybean	92	34	24	150
Grapevine	27	9	9	45
Cucumber	31	18	10	59
Canola	39	18	9	66

**Fig. 1:**
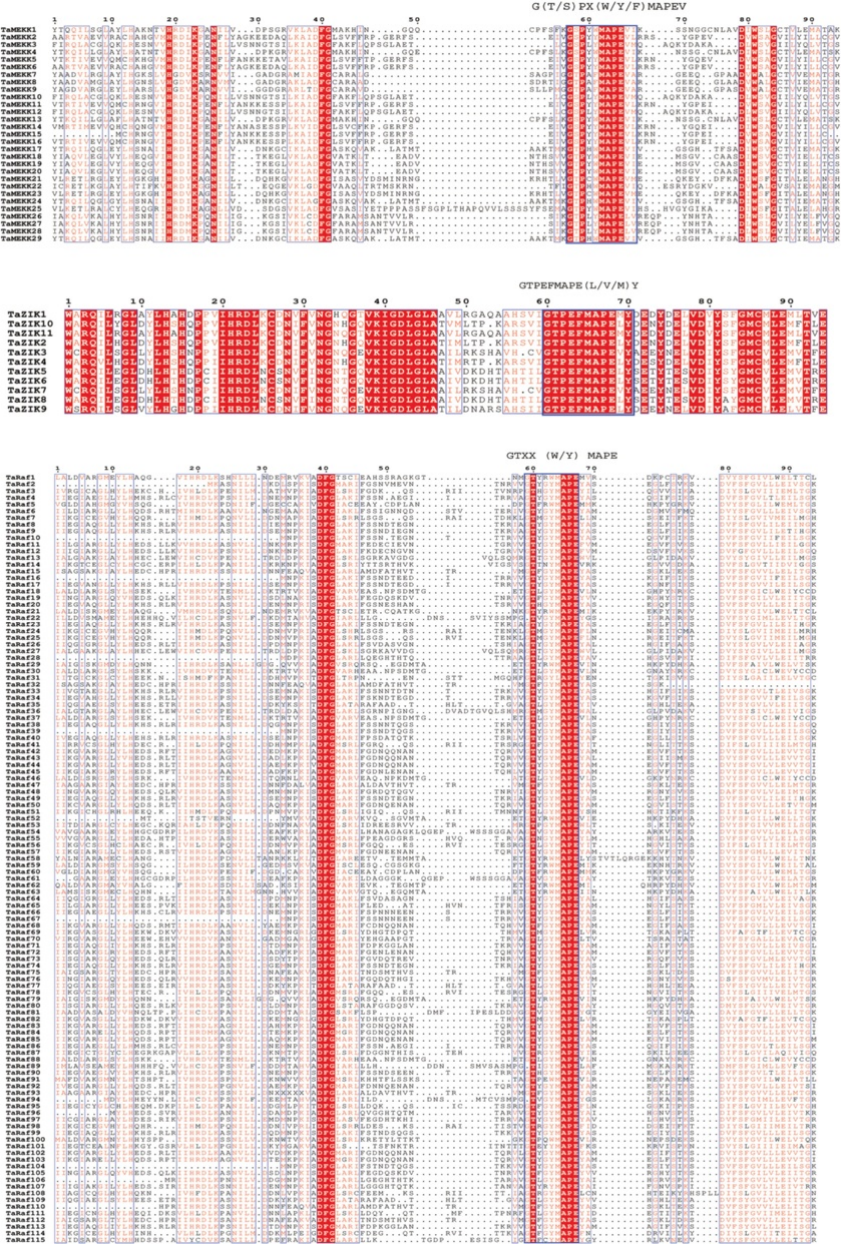
Protein sequence alignment of TaMAPKKK genes by ClustalW. The highlighted blue boxes showed the conserved signature motif

As reported in Arabidopsis and other plant species [12–15], the MAPKKK gene family could be subdivided into Raf, MEKK and ZIK subfamily according to the specific conserved signature motifs contained by these subfamilies, of which Raf had the signature of GTXX (W/Y) MAPE, ZIK of GTPEFMAPE (L/V) Y, and MEKK of G (T/S) PX (W/Y/F) MAPEV [15, 31]. To validate our prediction and subcategorize the identified wheat MAPKKKs, we further investigated the conserved signature motif in these TaMAPKKKs. Results showed that all the putative wheat MAPKKKs possessed at least one of the three conserved signature motifs (Fig. 1). Among them, 29 genes shared the conserved motif G (T/S) PX (W/Y/F) MAPEV, which were categorized into MEKK subfamily, and 11 had the motif GTPEFMAPE (L/V)Y, belonging to ZIK subfamily as well as the remaining 115 genes shared the motif GTXX (W/Y) MAPE, belonging to Raf subfamily. Then, we further named these gene based on the subfamily categories (Table 2). Moreover, the Raf subfamily is found to be the largest subfamily while the ZIK subfamily had the least members in wheat, which was consistent with the composition of MAPKKK genes in other species.

**Table 2:** Characteristics of the putative wheat MAPKKK genes

No.	MAPKKKs	Ensemble Wheat Gene ID	Subfamily	Subfamily Gene ID	Amino acid length	EST count	PI	MW (kDa)	Subcellular location	Location
1	TaMAPKKK1	Traes_2BL_23D01E7F4	MEKK	TaMEKK1	174	1	8.46	19.5	Extracellular PlasmaMembrane	scaffold_2BL_6949321:447-1269
2	TaMAPKKK2	Traes_4DS_63F7CF3CE		TaMEKK2	424	17	5.46	47.7	Cytoplasmic	scaffold_4DS_2304216:3-2906
3	TaMAPKKK3	Traes_4BL_A7AE389EE		TaMEKK3	654	20	6.33	72.0	Nuclear	scaffold_4BL_6901486:6-5409
4	TaMAPKKK4	Traes_6BL_93505FEAF		TaMEKK4	186	0	6.95	20.8	Cytoplasmic	scaffold_6BL_4252290:2480-4222
5	TaMAPKKK5	Traes_2AS_6DA49285E		TaMEKK5	424	95	5.95	48.2	Cytoplasmic	scaffold_2AS_5236692:1-3092
6	TaMAPKKK6	Traes_4BS_E01B5DAC9		TaMEKK6	398	18	5.94	44.9	Cytoplasmic	4B:9539577-9542587
7	TaMAPKKK7	TRAES3BF169900020CFD_g		TaMEKK7	473	4	4.64	49.8	Chloroplast	3B:24030208-24031629
8	TaMAPKKK8	TRAES3BF036800120CFD_g		TaMEKK8	431	1	5.13	46.1	Cytoplasmic Chloroplast	3B:452802187-452803479
9	TaMAPKKK9	TRAES3BF036800100CFD_g		TaMEKK9	366	5	4.55	38.2	Cytoplasmic Chloroplast	3B:452828028-452829181
10	TaMAPKKK10	Traes_4DL_94E10E6EB		TaMEKK10	659	21	6.44	72.5	Nuclear	4D:19445439-19451009
11	TaMAPKKK11	Traes_5DL_ADFFAE33D		TaMEKK11	450	36	5.84	51.1	Cytoplasmic	5D:146319049-146323269
12	TaMAPKKK12	Traes_4AS_DF85CBD39		TaMEKK12	710	21	6.55	77.7	Nuclear	4A:60064569-60070396
13	TaMAPKKK13	Traes_6AL_E854742BB		TaMEKK13	186	0	7.67	20.8	Cytoplasmic Extracellular	6A:166723325-166725190
14	TaMAPKKK14	Traes_5AS_9A8A9187C		TaMEKK14	404	22	5.32	45.9	Cytoplasmic	5A:52959512-52965983
15	TaMAPKKK15	Traes_5AL_DEDF36AD2		TaMEKK15	355	29	5.86	40.5	Cytoplasmic	5A:127609658-127614056
16	TaMAPKKK16	Traes_5BL_35A6B4387		TaMEKK16	557	29	5.95	62.7	Cytoplasmic	5B:250599335-250602791
17	TaMAPKKK17	Traes_5AL_4D0919BA1		TaMEKK17	549	9	5.7	60.9	Nuclear	scaffold_5AL_2767817:3993-8685
18	TaMAPKKK18	Traes_2BL_84B12F4F8		TaMEKK18	1262	47	5.86	139.6	Nuclear	scaffold_2BL_8013221:1461-11089
19	TaMAPKKK19	Traes_2DL_000136878		TaMEKK19	1267	44	5.69	139.8	Nuclear	2D:137763450-137774947
20	TaMAPKKK20	Traes_2AL_66079157A		TaMEKK20	1059	22	5.54	116.6	Nuclear	2A:238560833-238569155
21	TaMAPKKK21	Traes_6AS_E690A27CA		TaMEKK21	543	3	6.83	61.2	Cytoplasmic	6A:131214661-131219615
22	TaMAPKKK22	Traes_5AL_F9C2BEAF3		TaMEKK22	601	5	5.4	66.2	Cytoplasmic Nuclear	5A:109832378-109839192
23	TaMAPKKK23	Traes_6DS_185723D1E		TaMEKK23	480	3	6.59	54.6	Cytoplasmic Nuclear	6D:52694919-52699797
24	TaMAPKKK24	Traes_5BL_3EFFD8013		TaMEKK24	547	5	5.75	60.4	Nuclear	5B:45438771-45443053
25	TaMAPKKK25	Traes_5BL_38DB82ACF		TaMEKK25	518	0	6.01	56.5	Cytoplasmic Chloroplast	5B:75941978-75943867
26	TaMAPKKK26	Traes_2DS_122AEE879		TaMEKK26	1302	4	7.79	142.3	PlasmaMembrane	scaffold_2DS_5390089:1-10763
27	TaMAPKKK27	Traes_2BS_8506C57C5		TaMEKK27	1335	5	8.01	146.1	PlasmaMembrane	scaffold_2BS_1798276:2-10405
28	TaMAPKKK28	Traes_2AS_F0521C4F2		TaMEKK28	1332	5	8.09	145.9	PlasmaMembrane	2A:17064310-17075483
29	TaMAPKKK29	Traes_5DL_243735D6C		TaMEKK29	617	5	5.89	68.0	Cytoplasmic Nuclear	5D:48513467-48518535
30	TaMAPKKK30	Traes_5DL_9824E97A8	ZIK	TaZIK1	640	17	5.71	70.6	Nuclear	scaffold_5DL_4596034:10027-17090
31	TaMAPKKK31	Traes_6DL_F70F83614		TaZIK2	616	13	4.86	68.9	Cytoplasmic Nuclear	scaffold_6DL_3325277:1-4055
32	TaMAPKKK32	Traes_2AS_2B84A0A98		TaZIK3	650	33	5.56	72.9	Nuclear	scaffold_2AS_3354645:196-4869
33	TaMAPKKK33	Traes_6BL_4A17F7221		TaZIK4	617	13	4.89	69.0	Nuclear	scaffold_6BL_4289517:41-4156
34	TaMAPKKK34	Traes_2DS_AA3E486F3		TaZIK5	321	16	6.62	36.2	Cytoplasmic Nuclear	2D:43089164-43091159
35	TaMAPKKK35	Traes_2AS_E27D25DA3		TaZIK6	213	13	6.1	24.1	Cytoplasmic Nuclear	2A:69759079-69760992
36	TaMAPKKK36	Traes_2BS_18264AA5C		TaZIK7	703	32	5.61	78.6	Nuclear	2B:135976808-135980180
37	TaMAPKKK37	Traes_2BS_1E887CFE5		TaZIK8	292	13	6.1	33.0	Cytoplasmic	2B:157476501-157478662
38	TaMAPKKK38	Traes_1DS_34EFDA767		TaZIK9	243	3	5.91	27.9	Cytoplasmic	1D:3919344-3922775
39	TaMAPKKK39	Traes_6AL_48165ABE5		TaZIK10	616	13	4.82	68.9	Cytoplasmic Nuclear	6A:166642548-166647057
40	TaMAPKKK40	Traes_5BL_4002B5518		TaZIK11	640	17	5.55	70.5	Nuclear	5B:140747940-140754957
41	TaMAPKKK41	Traes_6DS_D8750EB5A	Raf	TaRaf1	326	3	8.65	36.6	Nuclear	scaffold_6DS_1052516:1426-2508
42	TaMAPKKK42	Traes_2BL_4CAF2C184		TaRaf2	149	7	5.07	16.5	Extracellular	2B:344488349-344489312
43	TaMAPKKK43	Traes_6BL_01E6CE316		TaRaf3	882	10	6	99.6	Cytoplasmic Nuclear	6B:192776834-192783783
44	TaMAPKKK44	Traes_2DS_DFE006BB6		TaRaf4	236	19	6.08	26.9	PlasmaMembrane	2D:2355728-2357164
45	TaMAPKKK45	Traes_3DL_CFCA7AA6B		TaRaf5	280	10	6.1	31.7	Cytoplasmic	scaffold_3DL_6928571:2813-4619
46	TaMAPKKK46	Traes_2DS_0BFF3B23D		TaRaf6	342	4	6.26	38.9	Cytoplasmic	2D:9025906-9028377
47	TaMAPKKK47	Traes_7DS_361EC0618		TaRaf7	454	0	5.3	50.8	Cytoplasmic Nuclear	7D:151974-158365
48	TaMAPKKK48	Traes_7DS_A3EB5BFEB		TaRaf8	272	19	5.82	30.8	PlasmaMembrane Cytoplasmic	7D:15224206-15225510
49	TaMAPKKK49	Traes_7DS_7A0BEA59B		TaRaf9	267	14	6.79	30.1	Cytoplasmic Chloroplast	7D:15301325-15302622
50	TaMAPKKK50	Traes_7DS_D56FBFFD4		TaRaf10	180	12	4.86	19.9	PlasmaMembrane	7D:19252002-19255310
51	TaMAPKKK51	Traes_7DS_5A97B2141		TaRaf11	177	5	5.25	20.1	Cytoplasmic	7D:44647285-44648284
52	TaMAPKKK52	Traes_7DS_342F25C32		TaRaf12	380	4	8.56	42.8	PlasmaMembrane	7D:87713571-87717063
53	TaMAPKKK53	Traes_1BL_C9B36DE76		TaRaf13	247	15	5.83	27.8	Cytoplasmic	1B:269260712-269261808
54	TaMAPKKK54	Traes_7DL_F0110933B		TaRaf14	714	17	6.28	79.7	Extracellular Cytoplasmic	7D:221995565-222000466
55	TaMAPKKK55	Traes_3DS_0694296CB		TaRaf15	199	33	6.2	22.1	Cytoplasmic	3D:812187-813154
56	TaMAPKKK56	Traes_3DS_4E61EE6EA		TaRaf16	180	16	4.94	20.0	PlasmaMembrane	3D:2782290-2783296
57	TaMAPKKK57	Traes_3DS_6801BD0D2		TaRaf17	279	33	5.24	31.3	PlasmaMembrane	3D:3073536-3075436
58	TaMAPKKK58	Traes_3DL_B28036C5B		TaRaf18	284	19	7.05	31.7	Cytoplasmic	3D:56193757-56197452
59	TaMAPKKK59	Traes_2AS_9219695D6		TaRaf19	340	6	5.89	37.4	Cytoplasmic Chloroplast	2A:121409421-121412207
60	TaMAPKKK60	Traes_2AS_79A94F84A		TaRaf20	229	1	6.44	26.1	PlasmaMembrane	2A:155554112-155555589
61	TaMAPKKK61	Traes_7DL_705BA7CDD		TaRaf21	218	3	9.24	24.9	Mitochondrial Nuclear	7D:60185604-60186553
62	TaMAPKKK62	Traes_4AL_1C557F688		TaRaf22	255	6	5.9	28.5	PlasmaMembrane Cytoplasmic	4A:171143548-171144835
63	TaMAPKKK63	Traes_4AL_06A8F8B8F		TaRaf23	287	13	7.19	32.5	PlasmaMembrane Cytoplasmic	4A:183127766-183129049
64	TaMAPKKK64	Traes_4AL_FEFC21AAB		TaRaf24	709	2	5.24	79.4	Cytoplasmic	4A:211420094-211424697
65	TaMAPKKK65	Traes_4AL_C217A20A1		TaRaf25	741	3	5.79	82.8	PlasmaMembrane Cytoplasmic	4A:211772709-211779190
66	TaMAPKKK66	Traes_1DL_FB90601E7		TaRaf26	348	5	6.76	30.5	Cytoplasmic Mitochondrial Nuclear	1D:93818790-93820691
67	TaMAPKKK67	Traes_1DL_F49D0E56A		TaRaf27	248	15	5.54	28.0	Cytoplasmic	1D:116551471-116552444
68	TaMAPKKK68	Traes_1DL_A0FB3E1D3		TaRaf28	193	14	5.14	21.7	Extracellular Cytoplasmic	1D:129495165-129496613
69	TaMAPKKK69	Traes_2DL_C5A0BDC60		TaRaf29	271	18	9.33	31.0	Mitochondrial Nuclear	2D:144590634-144593681
70	TaMAPKKK70	Traes_1DL_56B195A26		TaRaf30	289	25	7.49	31.9	Cytoplasmic Nuclear	1D:129622264-129624911
71	TaMAPKKK71	Traes_6AS_006C344A3		TaRaf31	786	6	5.89	90.0	Cytoplasmic Nuclear	6A:146084-152036
72	TaMAPKKK72	Traes_3AS_A2CECBF17		TaRaf32	243	30	6.34	26.9	Cytoplasmic Nuclear	3A:1529045-1530295
73	TaMAPKKK73	Traes_3AS_769E90DDD		TaRaf33	268	13	8.12	29.9	PlasmaMembrane	3A:4632011-4633193
74	TaMAPKKK74	Traes_3AS_5AF26B2FC		TaRaf34	327	10	6.72	36.8	PlasmaMembrane	3A:5100634-5102019
75	TaMAPKKK75	Traes_3AS_A542EC6F6		TaRaf35	305	8	7.21	34.3	Mitochondrial	3A:15435755-15437806
76	TaMAPKKK76	Traes_3AL_7F6E774BB		TaRaf36	253	11	5.27	28.3	Cytoplasmic	3A:91931309-91932151
77	TaMAPKKK77	Traes_3AL_943665768		TaRaf37	279	18	7.05	31.2	Cytoplasmic	3A:107041859-107044259
78	TaMAPKKK78	Traes_3AL_60BB7086F		TaRaf38	183	33	8.44	20.6	PlasmaMembrane Nuclear	3A:178617601-178618324
79	TaMAPKKK79	Traes_3AL_F384515F5		TaRaf39	188	24	4.81	21.0	Extracellular Cytoplasmic	3A:180162239-180164198
80	TaMAPKKK80	Traes_2AS_0C8932B8E		TaRaf40	339	7	5.54	38.8	Cytoplasmic Nuclear	2A:180067672-180069167
81	TaMAPKKK81	Traes_5AL_3FE725FD4		TaRaf41	775	2	6.28	88.0	Cytoplasmic Nuclear	5A:82903861-82912218
82	TaMAPKKK82	Traes_5AL_A236B0387		TaRaf42	259	11	5.49	29.2	Cytoplasmic	5A:96483013-96484223
83	TaMAPKKK83	Traes_5AL_CDD4A02E7		TaRaf43	299	5	6.36	33.8	PlasmaMembrane	5A:97062318-97064376
84	TaMAPKKK84	Traes_5AL_13784C39B		TaRaf44	233	6	5.46	26.3	PlasmaMembrane	5A:97195379-97196530
85	TaMAPKKK85	Traes_5AL_68C659562		TaRaf45	272	8	5.2	30.6	PlasmaMembrane	5A:99451668-99452790
86	TaMAPKKK86	Traes_5AL_7B1C0342F		TaRaf46	339	40	8.16	38.0	Extracellular PlasmaMembrane	5A:105814700-105817645
87	TaMAPKKK87	Traes_1AS_BEE845715		TaRaf47	388	18	6.32	43.0	Cytoplasmic Nuclear	1A:100519-103703
88	TaMAPKKK88	Traes_1AL_C21696173		TaRaf48	332	27	6.25	36.8	Nuclear	1A:243280434-243282190
89	TaMAPKKK89	Traes_7AS_51069274F		TaRaf49	264	17	6.13	29.8	Cytoplasmic	7A:12995054-12996342
90	TaMAPKKK90	Traes_7AS_81545C211		TaRaf50	214	3	8.93	24.0	Cytoplasmic Nuclear	7A:27180845-27181770
91	TaMAPKKK91	Traes_4DS_7D8A5F90B		TaRaf51	755	4	6.45	85.9	Cytoplasmic	4D:38444291-38457344
92	TaMAPKKK92	Traes_5DL_3191490FE		TaRaf52	160	50	7.02	18.0	Cytoplasmic	5D:119596889-119599696
93	TaMAPKKK93	Traes_5BS_0B466F42F		TaRaf53	278	0	7.59	31.8	Nuclear	5B:4053009-4053978
94	TaMAPKKK94	Traes_5BS_43731B6AC		TaRaf54	285	8	6.41	30.4	Cytoplasmic Chloroplast	5B:4123947-4125118
95	TaMAPKKK95	Traes_5BL_E44E042FD		TaRaf55	344	6	9.3	37.6	Nuclear	5B:106916097-106920463
96	TaMAPKKK96	Traes_5BL_2DA8896EE		TaRaf56	784	2	8.4	88.3	Cytoplasmic Nuclear	5B:178405794-178411614
97	TaMAPKKK97	Traes_5BL_11A7A1F5C		TaRaf57	205	9	9.3	23.2	Cytoplasmic	5B:206004103-206004989
98	TaMAPKKK98	Traes_5DL_294C4EDB3		TaRaf58	387	49	7.58	42.2	Nuclear	5D:148108984-148113098
99	TaMAPKKK99	Traes_3AS_2A0765E10		TaRaf59	279	29	8.13	31.2	PlasmaMembrane	3A:671046-672777
100	TaMAPKKK100	Traes_3AL_82306B917		TaRaf60	316	9	6.82	35.9	Cytoplasmic	3A:154206856-154208804
101	TaMAPKKK101	Traes_5DS_53F8C78FA		TaRaf61	199	8	6.01	21.1	Cytoplasmic	5D:10503237-10504290
102	TaMAPKKK102	Traes_7BL_46880A4FE		TaRaf62	280	119	8.7	31.5	Mitochondrial	scaffold_7BL_6485684:8-1478
103	TaMAPKKK103	Traes_7AL_9AD23808D		TaRaf63	314	2	6.9	35.4	Cytoplasmic	7A:84246015-84251550
104	TaMAPKKK104	Traes_1DL_0162A6BAC		TaRaf64	241	7	5.98	26.8	Cytoplasmic Nuclear	scaffold_1DL_2275852:3-2035
105	TaMAPKKK105	Traes_3AS_A0EA6D12C		TaRaf65	210	7	6.08	24.0	Cytoplasmic Mitochondrial Nuclear	scaffold_3AS_1117810:1-1084
106	TaMAPKKK106	Traes_4AL_48E7FB1C6		TaRaf66	197	11	6.15	22.5	PlasmaMembrane	scaffold_4AL_7145827:1-952
107	TaMAPKKK107	Traes_4AL_83D9333FE		TaRaf67	154	9	6.82	17.4	PlasmaMembrane	scaffold_4AL_7109061:3-710
108	TaMAPKKK108	Traes_5DL_62B6846F6		TaRaf68	191	7	6.3	21.7	PlasmaMembrane Cytoplasmic	scaffold_5DL_4605280:630-1568
109	TaMAPKKK109	Traes_2DS_42A9CC22D		TaRaf69	252	3	5.24	27.9	Cytoplasmic	scaffold_2DS_838920:50-1605
110	TaMAPKKK110	Traes_4BL_3626CDB73		TaRaf70	265	1	5.61	28.8	Cytoplasmic	scaffold_4BL_7036128:2-919
111	TaMAPKKK111	Traes_3AL_5DC02A5FC		TaRaf71	302	7	6.14	33.3	Cytoplasmic Chloroplast	scaffold_3AL_1833470:519-2133
112	TaMAPKKK112	Traes_5DL_0A74AE348		TaRaf72	297	5	5.76	33.5	PlasmaMembrane	5D:124050225-124051615
113	TaMAPKKK113	Traes_3AS_C492FCE9A		TaRaf73	242	3	6.52	27.1	Nuclear	scaffold_3AS_2578257:98-1277
114	TaMAPKKK114	Traes_4AL_32D968595		TaRaf74	270	17	6.1	30.5	Cytoplasmic	scaffold_4AL_7089761:892-2199
115	TaMAPKKK115	Traes_3AL_0187ECBAC		TaRaf75	159	7	5.39	17.9	Cytoplasmic Chloroplast	scaffold_3AL_4340950:1-1036
116	TaMAPKKK116	Traes_1BL_1E2841006		TaRaf76	267	19	6.24	30.2	Extracellular Cytoplasmic Nuclear	scaffold_1BL_3793082:882-2495
117	TaMAPKKK117	Traes_3DS_0B1914F50		TaRaf77	305	9	6.9	34.3	Cytoplasmic Mitochondrial	scaffold_3DS_2550735:71-2194
118	TaMAPKKK118	Traes_5DL_5DAC7A4CF		TaRaf78	497	3	5.88	56.4	Cytoplasmic	scaffold_5DL_4513923:4360-10186
119	TaMAPKKK119	Traes_2AL_0E43EBBB6		TaRaf79	180	13	7.06	20.3	Mitochondrial	scaffold_2AL_6381182:1-1586
120	TaMAPKKK120	Traes_4AL_9601B9873		TaRaf80	314	4	6.96	34.7	Nuclear	scaffold_4AL_7096965:1880-5803
121	TaMAPKKK121	Traes_2DS_964FA3D25		TaRaf81	245	13	4.64	27.1	Cytoplasmic	scaffold_2DS_5355140:3031-4467
122	TaMAPKKK122	Traes_2AS_DCD2F10331		TaRaf82	311	9	6.23	34.8	Cytoplasmic	scaffold_2AS_2039357:2956-4095
123	TaMAPKKK123	Traes_5DL_A367964F5		TaRaf83	225	10	8.79	25.2	Cytoplasmic	5D:124089352-124090277
124	TaMAPKKK124	Traes_2AS_AC9886ABC		TaRaf84	225	12	8.88	25.3	Cytoplasmic Nuclear	scaffold_2AS_5255912:5418-6352
125	TaMAPKKK125	Traes_7DS_81C827CE6		TaRaf85	363	4	6.27	40.5	PlasmaMembrane Cytoplasmic	scaffold_7DS_3862762:1862-7469
126	TaMAPKKK126	Traes_6BS_511AB47D71		TaRaf86	339	19	5.59	38.1	PlasmaMembrane Cytoplasmic	scaffold_6BS_3043664:2-1698
127	TaMAPKKK127	Traes_6DL_7662129AC		TaRaf87	928	55	5.77	104.3	Cytoplasmic Nuclear	scaffold_6DL_3324907:1786-5987
128	TaMAPKKK128	Traes_1BL_CDC566E72		TaRaf88	289	25	7.97	32.0	Cytoplasmic Nuclear	scaffold_1BL_3828880:5213-7383
129	TaMAPKKK129	Traes_6BL_658AE8589		TaRaf89	280	1	5.7	31.6	Cytoplasmic	scaffold_6BL_4262535:303-3102
130	TaMAPKKK130	Traes_7AS_0BE0D89AC		TaRaf90	251	14	5.79	28.6	PlasmaMembrane Cytoplasmic	scaffold_7AS_4255305:1753-2961
131	TaMAPKKK131	Traes_6BS_EAABDE59A		TaRaf91	250	47	9.14	28.4	Extracellular Mitochondrial	scaffold_6BS_3021108:276-3989
132	TaMAPKKK132	Traes_5BL_17A56822E		TaRaf92	221	6	7.69	24.8	PlasmaMembrane Cytoplasmic	scaffold_5BL_10894314:6618-8227
133	TaMAPKKK133	Traes_1BS_EA26D2661		TaRaf93	388	18	6.32	42.5	Cytoplasmic Nuclear	scaffold_1BS_3482116:8155-10572
134	TaMAPKKK134	Traes_5DL_383D5A71F		TaRaf94	189	11	5.94	21.0	PlasmaMembrane Nuclear	5D:157768052-157768754
135	TaMAPKKK135	Traes_2DL_77990F25A		TaRaf95	319	1	7.11	36.4	Cytoplasmic Nuclear	scaffold_2DL_9829349:7066-8506
136	TaMAPKKK136	Traes_2BS_C0AED9734		TaRaf96	219	2	4.72	24.5	Cytoplasmic Nuclear	scaffold_2BS_5191771:1720-2933
137	TaMAPKKK137	Traes_3DL_73ACAB95C		TaRaf97	309	9	6.14	34.8	Cytoplasmic Nuclear	scaffold_3DL_6924167:1792-4345
138	TaMAPKKK138	Traes_7DS_03068057C		TaRaf98	259	0	7.07	29.6	Cytoplasmic Nuclear	scaffold_7DS_3924816:112-1661
139	TaMAPKKK139	Traes_3AL_AB54706CA		TaRaf99	381	26	5.69	43.1	Cytoplasmic Nuclear	scaffold_3AL_4360739:391-3058
140	TaMAPKKK140	Traes_5BS_F1687AA56		TaRaf100	231	30	9.33	27.1	Mitochondrial	scaffold_5BS_2278981:2727-5793
141	TaMAPKKK141	Traes_7DS_A46AFAE10		TaRaf101	918	5	6.62	102.6	PlasmaMembrane Cytoplasmic	scaffold_7DS_3809424:2024-7790
142	TaMAPKKK142	Traes_2AS_CC27D1C41		TaRaf102	248	8	7.64	27.8	Cytoplasmic	scaffold_2AS_5226094:20239-21469
143	TaMAPKKK143	Traes_2AS_AC9886ABC1		TaRaf103	225	12	8.88	25.3	Cytoplasmic Nuclear	scaffold_2AS_5255913:5418-6352
144	TaMAPKKK144	Traes_3DL_3D1CAD68F		TaRaf104	188	15	4.84	20.9	Cytoplasmic	scaffold_3DL_6944830:139-1513
145	TaMAPKKK145	Traes_2BS_5C64FC44A		TaRaf105	265	11	6.33	29.8	Cytoplasmic	2B:125675753-125677190
146	TaMAPKKK146	Traes_4BS_C5AB35B0C		TaRaf106	203	10	5.84	22.6	Mitochondrial Chloroplast	scaffold_4BS_948180:48-952
147	TaMAPKKK147	Traes_2AS_E5AB3458C		TaRaf107	347	3	6.57	39.6	Nuclear	scaffold_2AS_5232094:4234-6292
148	TaMAPKKK148	Traes_1BS_41E5F1990		TaRaf108	269	6	6.09	30.9	Cytoplasmic	scaffold_1BS_3451546:6832-8016
149	TaMAPKKK149	Traes_3B_582DCEA06		TaRaf109	352	8	7.74	39.2	Cytoplasmic Mitochondrial	scaffold_3B_10637137:56-2229
150	TaMAPKKK150	TRAES3BF061500080CFD_t1		TaRaf110	340	30	5.29	37.6	Cytoplasmic Nuclear	3B:1864715-1866712
151	TaMAPKKK151	TRAES3BF104900080CFD_t1		TaRaf111	1005	9	6.67	111.9	Nuclear	3B:97278846-97291325
152	TaMAPKKK152	TRAES3BF026200090CFD_t1		TaRaf112	396	9	6.24	43.7	Cytoplasmic	3B:421410785-421414323
153	TaMAPKKK153	TRAES3BF086600060CFD_t1		TaRaf113	302	8	6.25	33.4	Cytoplasmic Mitochondrial	3B:552717475-552718658
154	TaMAPKKK154	TRAES3BF078400040CFD_t1		TaRaf114	775	3	5.67	87.6	PlasmaMembrane Cytoplasmic Nuclear	3B:696462241-696470991
155	TaMAPKKK155	Traes_6BS_5BFDC774A		TaRaf115	318	2	5.2	36.1	PlasmaMembrane	6B:84413110-84414856

To support the actual existence of these wheat MAPKKKs, we further performed a BLASTN search against the wheat expressed sequence tag (EST) and unigene database using the MAPKKKs as query. Results showed that most of the TaMAPKKKs’ existences were supported by EST hits except 6 MAPKKKs (TaMEKK4, TaMEKK13, TaMEKK25, TaRaf7, TaRaf53 and TaRaf98). We speculated these 6 not-support TaMAPKKKs might not express under any the used conditions or express with very low level that cannot be detected experimentally. Among the supported TaMAPKKK genes, TaRaf62 has the largest hits of ESTs, with the number of 119, followed by TaMEKK5 and TaRaf87 with the number of 95 and 55 ESTs, respectively.

Chromosome localization analysis found that the 155 TaMAPKKK genes were unevenly distributed on all the 21 wheat chromosomes, of which chromosome 3A contained the most MAPKKK genes with the number of 15, followed by 2A with the number of 14, then 5B, 5D as well as 7D all with the number of 11, while the chromosome 7B had the least MAPKKK gene, with the number of only 1. Furthermore, the length of putative TaMAPKKK proteins ranged from 149 to 1335 amino acids, with the putative molecular weight (Mw) ranging from 16.5 to 146.1 kDa and theoretical isoelectric point (pI) ranging from 4.55 to 9.33, respectively. The subcellular localization analysis found that a total of 51 TaMAPKKKs localized in nuclear, 42 localized in cytoplasmic and 32 localized in plasma membrane, while the remaining were predicted to be located in chloroplast, mitochondrial and extra-cellular (Table 2).

### Phylogenetic and conserved domains analysis of TaMAPKKKs

**Fig. 2:**
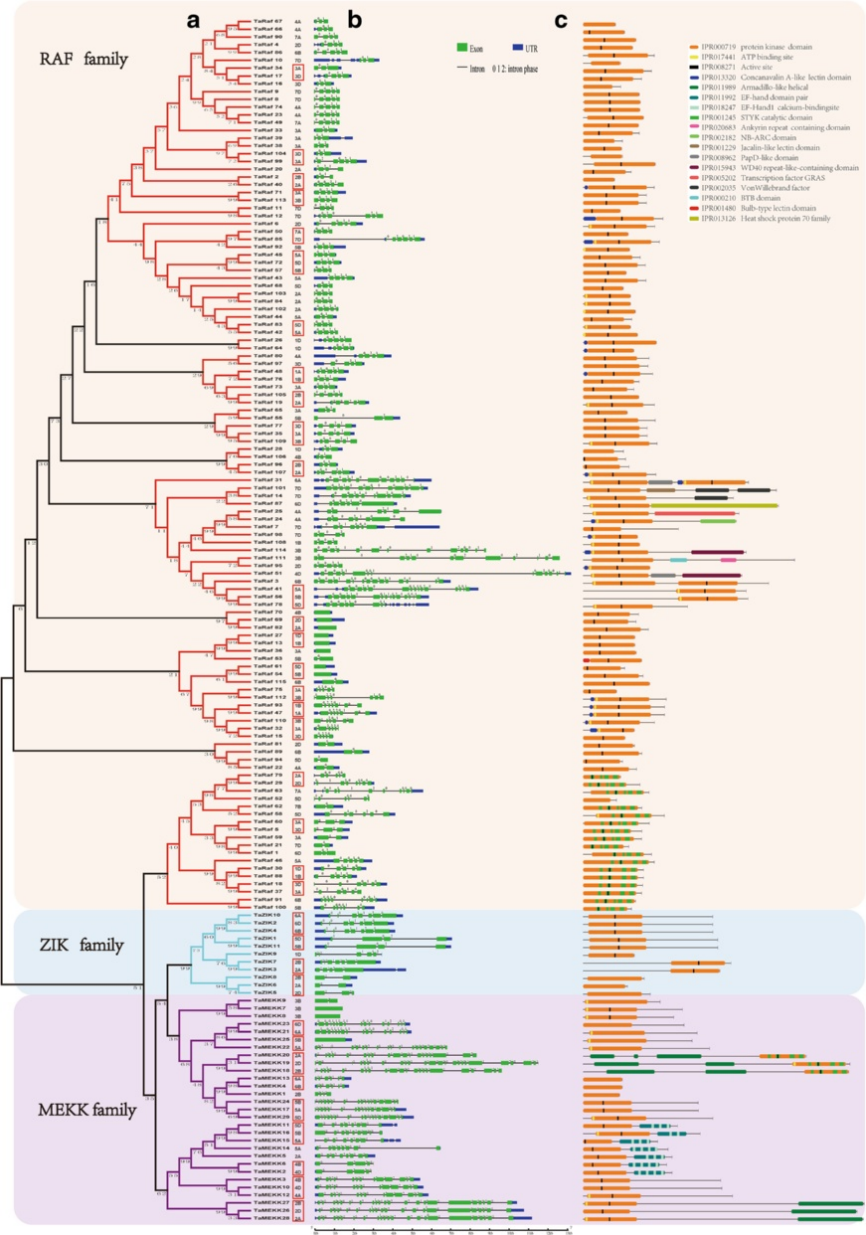
Phylogenetic relationships (**a**), gene structures (**b**) and protein structures (**c**) of MAPKKK genes in wheat

To further evaluate the phylogenetic relationships of the wheat MAPKKK cascade genes, the full-length protein sequences of the 155 TaMAPKKKs were aligned using ClustalW software and then the phylogenetic tree were constructed using the neighbor joining (NJ) method integrated into MEGA6.0 (Fig. 2a). On the basis of phylogenetic analysis, MAPKKKs in wheat were clustered into three major groups, of which MEKK, Raf and ZIK subfamily members clustered together into one category, respectively. It is found that the bootstrap value of the phylogenetic tree is low, which may due to the low similarity of the full-length protein sequences, suggesting that there are high sequence differentiation in these MAPKKK genes although the conserved motifs were included, which was consistent with the MAPKKKs in maize [12], rice [13] and Brachypodium [15, 32]. The conserved domains and phylogenetic relationship suggested that MAPKKK genes showing the closer phylogenetic relationship may have the similar biological function. To date, there is no report regarding MAPKKK genes in *T. aestivum*, so searching for MAPKKK family genes and understanding their phylogenetic relationship in *T. aestivum* is necessary and helpful for their further functional study.

Furthermore, the protein domains of these wheat MAPKKK genes were identified by searching against InterProScan databases (Fig. 2c). Results found that each cluster of the MAPKKKs classified by phylogenetic analysis shared the similar protein structure and domain composition, demonstrating that the protein architecture is remarkably conserved within a specific subfamily of MAPKKKs. Protein kinases have been demonstrated to play the crucial role in mediating process of protein phosphorylation, which widely occurred in most cellular activities [32]. In this study, we found all the TaMAPKKK proteins contained a kinase domain (IPR000719), and most of them had the serine/threonine protein kinase active site (IPR008271) in the central part of the catalytic domain. These features were also found in the MAPKKK proteins of rice and cucumber [13, 33], suggesting the conserved function of MAPKKK genes in plants. Moreover, the ATP-binding site, which is located on the catalytic domain, is the most conserved sequences in the kinase family [33]. We found that most of TaMAPKKKs also contained an ATP-binding site (IPR017441), suggesting that these wheat MAPK cascade kinases use ATP as the ligand in signal transduction pathway. In addition, the TaMAPKKKs also had some other conserved domains, such as concanavalin A-like lectin/glucanase domain (IPR013320), armadillo-like helical (IPR011989), and EF-hand domain (IPR011992). Interestingly, these TaMAPKKKs containing the same protein domains were generally clustered into the same clade in phylogenetic analysis, and showed similar expression patterns in response to multiple stresses, which was consistent with the result of BdMAPKKK genes as reported previously [32]. For example, most TaMAPKKK genes containing concanavalin A-like lectin/glucanase domain were up-regulated by drought stress, while those genes containing armadillo-like helical domain showed to be down-regulated under salt stress. These results indicated that the various protein domains could regulate the TaMAPKKK gene to exhibit specific biological functions. The conserved domains identification and analysis may facilitate the identification of functional units in these kinase genes and accelerate to understand their crucial roles in plant growth and development as well as stresses response [34, 35].

### Analyses of gene structures and promoter regions of TaMAPKKKs

Gene structure analysis can provide important information about the gene function, organization and evolution [36]. Thus, the exon/intron structures of TaMAPKKK genes were further analyzed using the available wheat genome annotation information and then were displayed by the Gene Structure Display Server (http://gsds.cbi.pku.edu.cn/) (Fig. 2b). We found the exon/intron structures in the TaMAPKKK genes were relatively conserved within the subfamily but some divergent between different subfamily. The Raf and MEKK subfamily have more sophisticated structure than ZIK subfamily due to the various number of intron. In detail, all the ZIK genes had introns, with the number ranging from 1 to 7. In the MEKK subfamily, 3 gene had no intron, and others had 1 to 22 introns, which was the most highly variable in the number of introns in TaMAPKKKs. In the Raf subfamily, 7 out 115 genes had no intron, and other Raf genes had the intron number ranging from 1 to 14. Interestingly, most gene pairs clustered together by phylogenetic analysis shared the similar exon/intron structure and intron phases in these TaMAPKKK genes, suggesting the evolutionary event may impact not only on the gene function but also on gene structure. It has been revealed that intron gain or loss is the results of selection pressures during evolution in plants, and the genes tend to evolve into diverse exon-intron structures and perform differential functions [37, 38]. Accordingly, the wheat MAPKKK genes were found to have the similar exon-intron structure within same subfamily, while the numbers of introns were varied, even within subfamily, which indicated that gene differentiation have occurred in the wheat MAPKKK to accomplish different biological functions under the selection pressure during the wheat genome formation and evolution.

Promoter is the region of the transcription factors (TF) binding site to initiate transcription, which plays a key role in regulating gene spatial and temporal expressions [39]. To further detect the possible biological function and transcription regulation of these TaMAPKKKs, the 2 kb-upstream region of the transcriptional start site of all these genes were extracted and then used to screen for cis-regulatory elements. Results showed that a large number of stress-related and hormone-related cis-elements were found in promoter regions of the wheat MAPKKK genes (Additional file 3), which were similar with the result in Brachypodium, tomato and cucumber [32, 33, 36]. In addition, the abiotic stress-related (a total of 9 drought-stress, 1 salt-stress, 1 heat-stress, 1 cold-stress, 2 wound-stress and 2 disease resistance-related) and hormones signaling transduction-related (6 gibberellins, 4 abscisic acid and 3 ethylene-related) cis-regulatory elements were also found, suggesting that the wheat MAPKKKs may involve in regulating varieties of stress responses and hormone signaling transduction processes.

### Genomic distribution and gene duplication of TaMAPKKK gene family

**Fig. 3:**
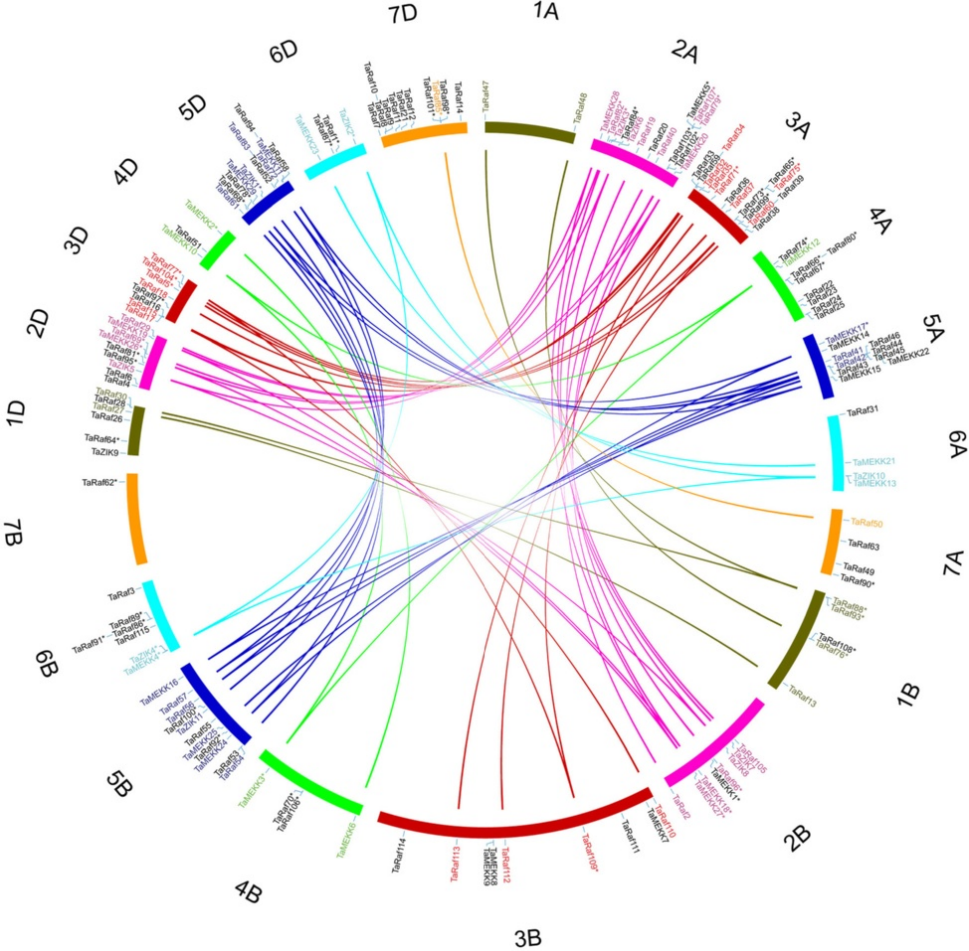
Chromosomal localization and the homologous TaMAPKKK genes in wheat A, B and D sub-genomes. The genes followed by * represent that the gene only anchor to scaffold. Seven homologous groups of wheat chromosomes are displayed in different colors. Duplicated genes of each homo-group are displayed in corresponding color and linked using lines with corresponding color

Based on the available wheat genome annotation information, the chromosomal location of the TaMAPKKK genes were further investigated (Fig. 3). A total of 58, 45, and 52 TaMAPKKK genes are distributed in the A, B and D sub-genome, respectively (A > D > B). Initial gene loss may occurred in B genomes following tetraploidy to decrease functional redundancy and define the core wheat genes, with subsequent loss from all three genomes following the formation of the hexaploid around 9000 years ago. The distribution of MAPKKK genes was not random in wheat chromosomes. There were 13, 31, 32, 16, 32, 15 and 16 genes in the group 1 to 7 chromosomes, which show two obvious gradients between group 2, 3, 5 and other four groups. And chromosome 3A had the highest number of MAPKKK genes with the value of 15 genes, whereas chromosome 7B had only one MAPKKK gene. These results indicates that duplication events of MAPKKK gene have likely occurred in wheat 2, 3 and 5 group chromosomes during wheat formation and the evolution of gene families within the different sub-genome is independent, which may associate with gene functions.

Gene duplication is frequently observed in plant genomes, arising from polyploidization or through tandem and segmental duplication associated with replication [40]. In our study, a total of 11 homologous gene groups with a copy on each of A, B and D homologous chromosome were found in wheat MAPKKK gene family, and 24 gene pairs with a copy on only 2 of the 3 homologous chromosomes were also identified (Fig. 3 and Additional file 4), while the remaining 74 genes were not found homologs in wheat genome. Previous studies have demonstrated that the fractionation from ploidy caused the loss of some homologous sequences because of some combination of deletion [41]. Our results indicated gene loss may also occur in wheat MAPKKK gene family, resulting in the loss of some homologous copies. The specific retention and dispersion of MAPKKKs in homologous chromosomes provide the invaluable information to better understand the wheat chromosome interaction and polyploidization. Furthermore, these homologous genes are clustered in group 2, 3 and 5 chromosomes, which was consistent with the above chromosome localization analysis, suggesting that group 2, 3 and 5 chromosomes suffered less sequence loss and interaction impact compared to other homologous chromosome groups.

**Fig. 4:**
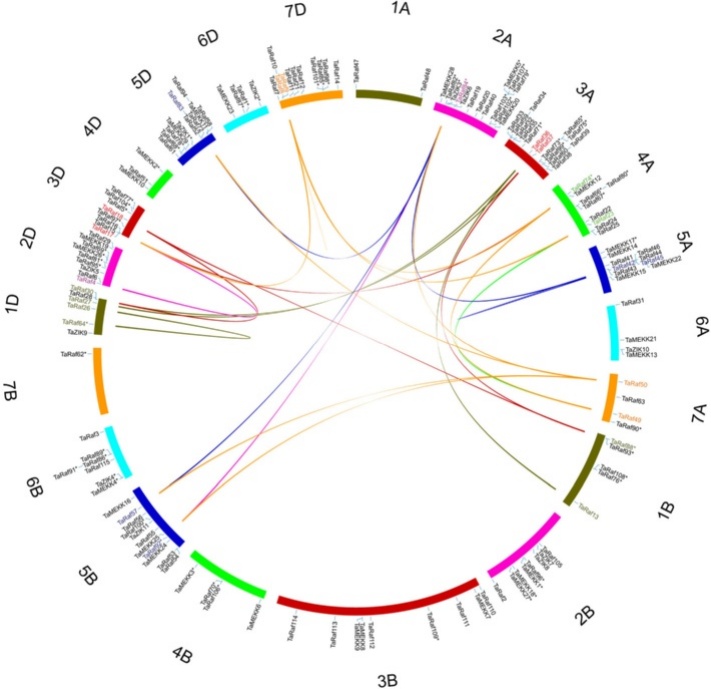
Duplicated MAPKKK genes pairs identified in wheat. Seven homologous groups of wheat chromosomes are displayed in different colors. Duplicated gene pairs are displayed in corresponding color and linked using lines with the corresponding color

Additionally, 25 pairs of duplication genes from different sub-genomes were also identified (Fig. 4 and Additional file 4), including 3 duplication events within the same chromosome and 22 segmental duplication events between different chromosomes, suggesting that the duplication events could play vital roles in the expansion of the MAPK cascade kinase genes in wheat genome. Interestingly, most duplication events occurred between A and D genomes, except the pair of Raf92 and Raf57 occurred on 5B as well as that of Raf13 and Raf88 from 1B. We postulated that the gene family size of the A and B sub-genome have arrived to balance after first hybridization with the long evolutionary process, but the D sub-genome, which was added to form hexaploid wheat recently, appeared to have more interaction with other two sub-genomes. More interestingly, all the 25 pairs of duplication genes belonging to Raf subfamily, which indicates that gene duplication is a main processes responsible for expanding family size and protein functional diversity [42].

### Regulatory network between TaMAPKKK genes with other wheat genes

**Fig. 5:**
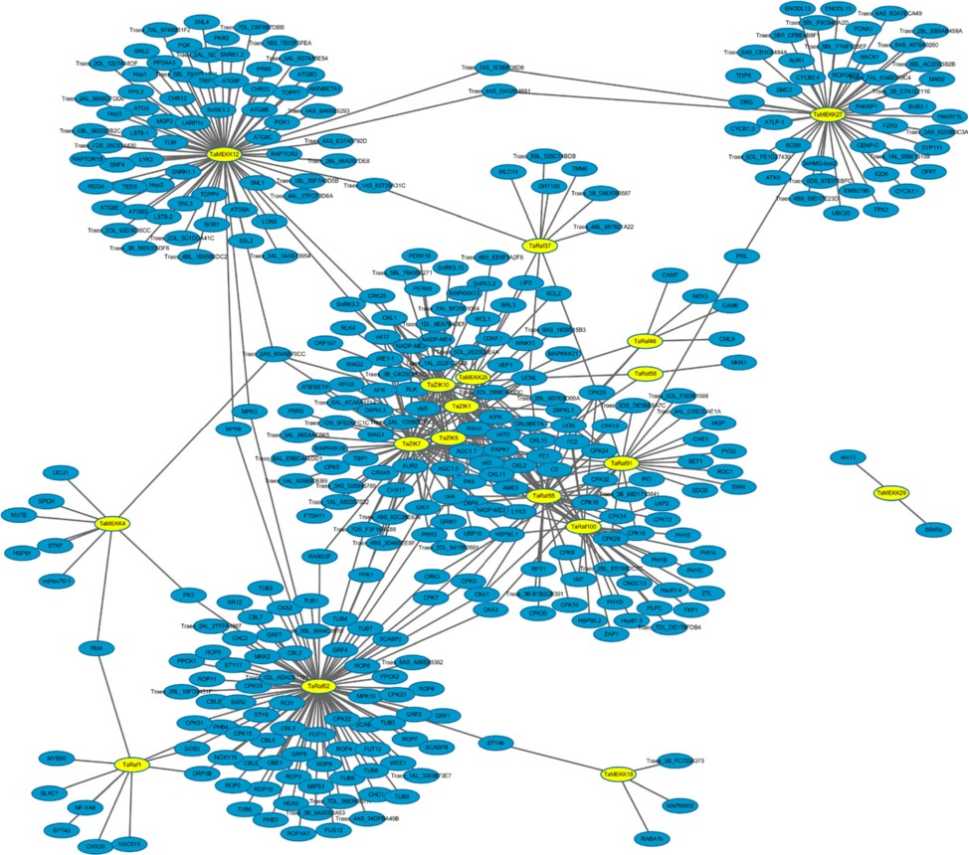
The interaction network of TaMAPKKK genes in Wheat according to the orthologs in Arabidopsis

**Fig. 6:**
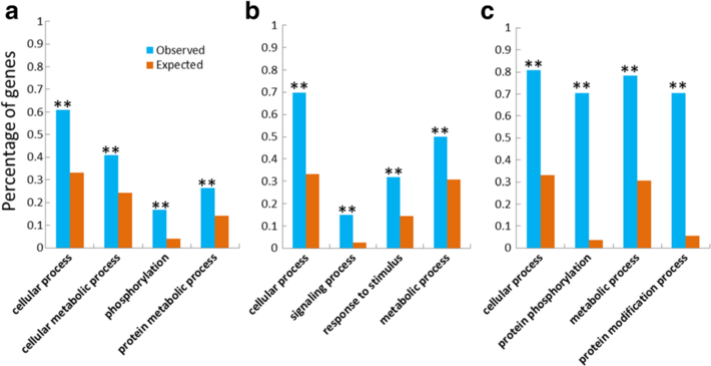
Functional categories of genes in MEKK (**a**), Raf (**b**), and ZIK (**c**) subfamily. FDR-adjusted P values, **P < 0.01, respectively. Observed, numbers of genes observed in this study; Expected, numbers of genes in this same category in the GO enrichment analysis program

MAPKKKs, as the first step of MAPK cascade, function as the pivotal component linking upstream signaling steps to the core MAPK cascade and then promote the corresponding cellular responses, which are activated by a diversity of external stimuli and interact with other genes to form the signaling regulatory network in plants [2, 31]. To understand the interactions between TaMAPKKKs and other wheat genes, the regulatory network of them (Fig. 5) was predicted using the orthology-based method [43]. Results showed 18 MAPKKKs (6 TaMEKKs, 8 TaRafs and 4 TaZIKs) were found to have homology with Arabidopsis genes, and corresponding 509 gene pairs of network interactions were detected with the average of 28.3 gene/TaMAPKKK, suggesting the MAPKKKs were widely involved in the regulatory network and metabolic processes in wheat (Additional files 5 and 6). Among them, 149 genes were interacted by TaZIKs, and 212 genes were interacted by TaRafs, as well as 148 genes interacted by TaMEKKs, respectively. TaMEKK27 showed orthologous to Arabidopsis Fused (FU) gene, with an active kinase domain and the C-terminal ARM/HEAT repeat domain. Previously study has revealed that Arabidopsis Fused kinase termed TIO is essential for cytokinesis in both sporophytic and gametophytic cell types [44]. In this study, TaMEKK27 was found to interact with 38 wheat genes, including SOS6, NACK1 and FZR3, suggesting it was also mainly involved in cell proliferation and cytokinesis. TaRaf1 is found to interact with 10 wheat genes, which is homology with Arabidopsis HT1 gene reported to encode an important protein kinase for regulation of stomatal movements and corresponding to CO2, ABA and light [45]. The predicted upstream target genes of TaRaf1 included SLAC1, FMA and CHX20 as well as MYB and NAC transcription factor, which indicated TaRaf1 might play a vital role in ion homeostasis and stress response in wheat. Furthermore, Gene Ontology (GO) functional enrichment of those genes was performed to understand their potential functions. GO descriptions of those interacted genes were involved in diverse biological process, molecular function and stress response. TaMEKK interacted genes were significantly enriched for cellular process and metabolic process, and TaRaf interacted genes were significantly enriched for cellular process and pathways for stress response, while TaZIK interacted genes were functionally enriched in cellular process and protein modification process pathway (Fig. 6a–c), which indicated that TaMAPKKK genes played the vital role in cellular response to external stimuli, especially TaRaf subfamily genes might be the main adaptors to transduce the stress-related signal.

### Tissue-specific expression patterns of TaMAPKKK genes

**Fig. 7:**
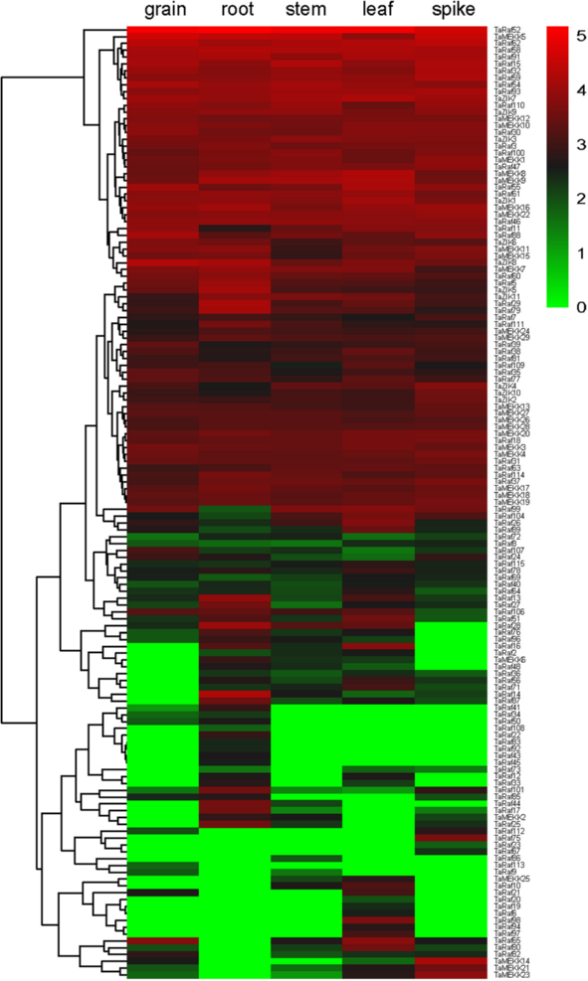
Hierarchical clustering of the expression profiles of all TaMAPKKK genes in five different organs or tissues (grain, root, stem, leaf and spike). Log10-transformed (FPKM + 1) expression values were used to create the heat map. The red or green colors represent the higher or lower relative abundance of each transcript in each sample

Different members of gene families exhibit great disparities in abundance among different tissues to accommodate different physiological processes [46, 47]. To gain insight into the temporal and spatial expression patterns and putative functions of MAPKKK genes in wheat growth and development, the tissue specificity of the 155 TaMAPKKK genes was investigated using available RNA-seq data for five different tissues [48]. Based on the log10-transformed (FPKM + 1) values, we found that the expression levels of the TaMAPKKKs varied significantly in different tissues (Fig. 7). Most MAPKKK genes were found to be expressed in at least one detected organ. All the members in ZIK subfamily were expressed in all of the 5 organs, while a total of 16 Raf genes had too weak expression abundances to be detected in any tissues, which indicated that these genes have undergone functional differentiation and redundancy. Most of MAPKKK genes were much more highly expressed in the root and leaf compared to grain, stem and spike. Furthermore, the tissue-specific expressed MAPKKK genes were identified. A total of 1, 6, 1, 6 and 3 genes were found to be specifically expressed in grain, root, stem, leaf and spike, respectively. Among them, TaRaf112 was predominantly expressed in grain and spike, TaMEKK25 showed preferential expression in stem and leave, and TaRaf12, TaRaf33 as well as TaRaf73 showed preferential expression in root and leave. As shown in Fig. 7 and Additional file 7, most homologous and duplication genes showed similar expression pattern during development. However, it also should be noted that many clustering of expression profiles does not reflect gene similarities, including the copies of one MAPKKK gene from sub-genomes and duplication genes from different sub-genomes. Some of them even show converse expression patterns. For instance, TaRaf71 which located in 3A showed preferential expression patterns in the root, stem, leaf and spike, whereas its homology gene TaRaf113 from 3B was only expressed in the grain. TaMAPKKK23 in 5A was expressed in all tested organs with relatively higher abundance, while its homology TaMAPKKK25 from 5B only slightly expressed in stem and leaf. The divergences in expression profiles between homologous genes revealed that some of them may lose function or acquire new function after polyploidy and duplication in the wheat evolutionary process.

### Expression patterns of TaMAPKKK genes under abiotic stresses

**Fig. 8:**
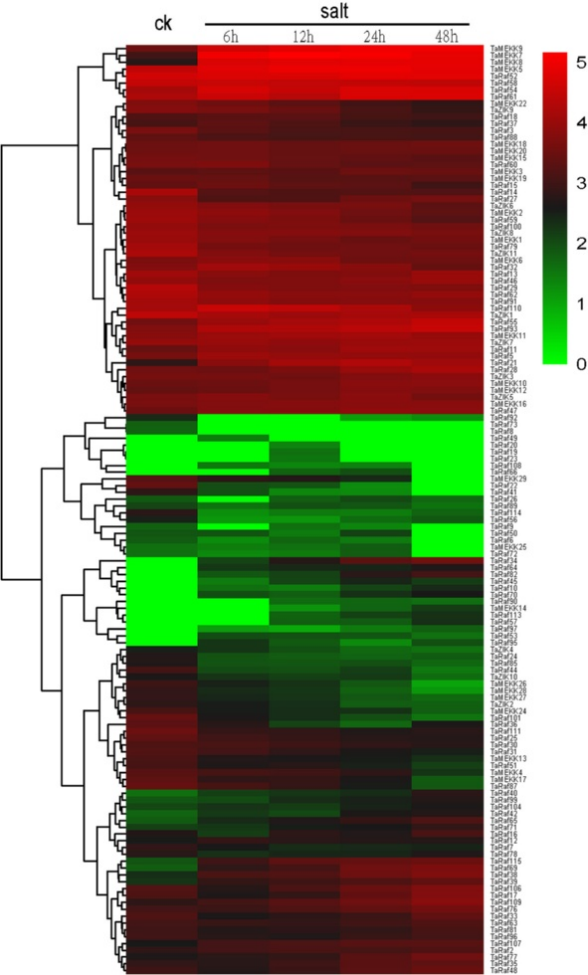
Hierarchical clustering of the expression profiles of all 155 TaMAPKKK genes under salt stress treatments. Log10-transformed (FPKM + 1) expression values were used to create the heat map. The red or green colors represent the higher or lower relative abundance of each transcript in each sample. Fold change cutoff of two and p-value < 0.05, q-value < 0.05 were taken as statistically significant

**Fig. 9:**
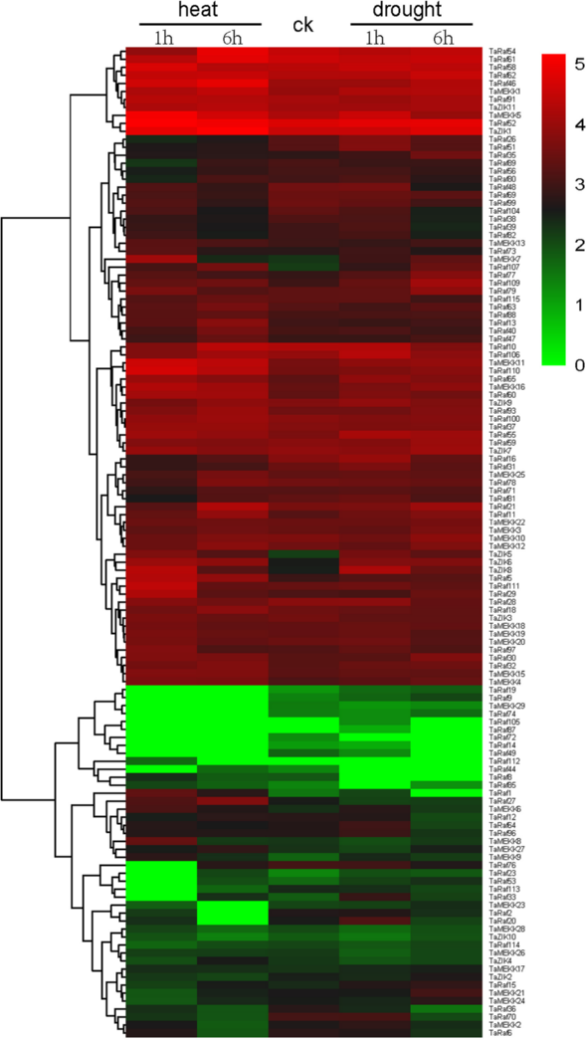
Hierarchical clustering of the expression profiles of all TaMAPKKK genes under drought and heat stress treatments. Log10-transformed (FPKM + 1) expression values were used to create the heat map. The red or green colors represent the higher or lower relative abundance of each transcript in each sample. Fold change cutoff of two and p-value < 0.05, q-value < 0.05 were taken as statistically significant

**Fig. 10:**
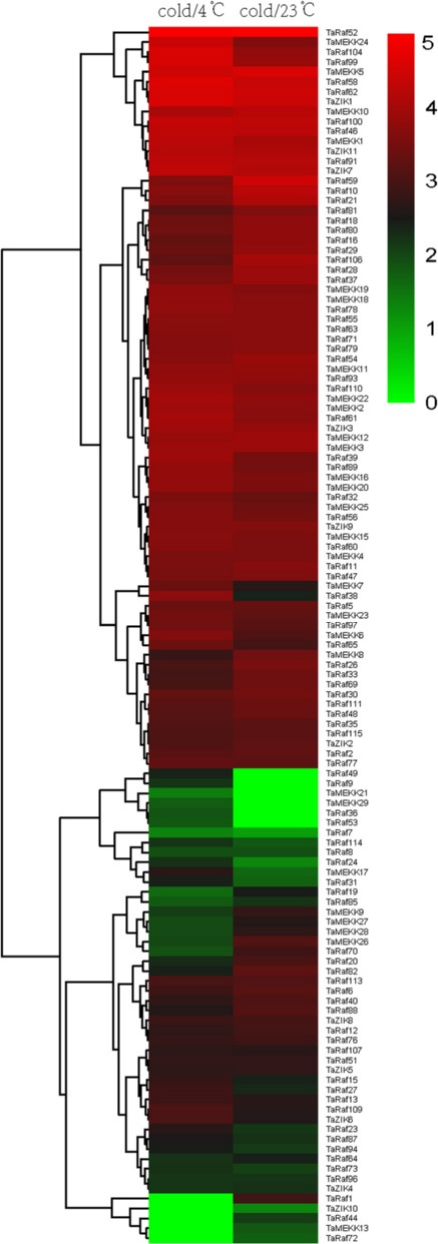
Hierarchical clustering of the expression profiles of all TaMAPKKK genes under cold stress treatments. Log10-transformed (FPKM + 1) expression values were used to create the heat map. The red or green colors represent the higher or lower relative abundance of each transcript in each sample. Fold change cutoff of two and p-value < 0.05, q-value < 0.05 were taken as statistically significant

Extensive studies have revealed that the MAPKKK genes played a crucial role in response to abiotic stresses in plant [10, 49, 50]. In the present study, expression patterns of all TaMAPKKK genes in response to four abiotic (salt, heat, drought, cold) stresses were investigated using RNA-seq data to study the roles of TaMAPKKK genes in the response to abiotic stresses. Overall, all the 155 wheat MAPKKK genes showed differential expression patterns under these conditions and most of them were up-regulated in response to more than one stress (Figs. 8, 9 and 10). Among them, TaMEKK14, TaRaf10, TaRaf34 and TaRaf53 showed specific-expression under salt stress, while TaRaf87 and TaRaf105 specifically expressed under drought stress. Meanwhile, TaRaf36 and TaRaf49 were specifically expressed under cold stress while TaRaf112 were specifically expressed under heat stress. In addition, some down-regulated TaMAPKKKs were also observed. TaMEKK29, TaRaf22, TaRaf41, and TaRaf73 was down-regulated under salt stress (Fig. 8), TaMEKK29 showing down-regulated under heat stress, while TaRaf44, TaRaf72 and TaRaf80 showing down-regulated under heat and drought stress (Fig. 9), as well as TaMEKK13, TaRaf1 and TaZIK10 were down-regulated under cold stress (Fig. 10), respectively. These stress-induced MAPKKK genes provided the valuable information to further reveal the roles of TaMAPKKKs playing in regulating wheat diverse stress processes. Finally, the most of the homologous and duplication gene pairs such as TaRaf110/TaRaf32/TaRaf15, and TaMEKK18/ TaMEKK19/ TaMEKK20 showed the similar expression pattern under these stress treatments, suggesting that these had similar physiological functions. On the other hand, several gene pairs such as TaRaf83/TaRaf42 and TaRaf17/TaRaf74, exhibited different expression patterns under the same stress treatments, suggesting functional differentiation has been occurred in these genes and they involved in regulating different stress signaling pathways.

### Validation of the expression of TaMAPKKKs by qRT-PCR analysis

**Fig. 11:**
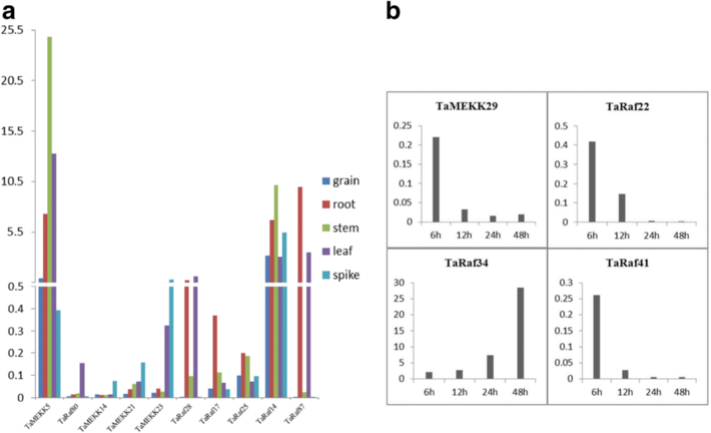
Validation of the expression level of TaMAPKKKs by qRT-PCR analysis. **a** The relative expression levels of the 10 selected TaMAPKKKs in different tissues; **b** The relative expression levels of the 4 TaMAPKKKs under salt treatment

Gene expression patterns usually provide the important clue for its function. Though expression profiles analysis based on RNA-seq data, the differentially expressed TaMAPKKKs among different tissues and stresses were obtained. To further verify the expression levels of these TaMAPKKKs, 10 differentially expressed genes in tissues and 4 salt-responsive genes were randomly selected to detect their expression levels through qRT-PCR analysis (Fig. 11). Among five tissues, TaMEKK5 was found to be expressed in all tested materials with relatively higher abundance. TaMEKK14, TaMEKK21 and TaMEKK23 were found to show a relatively high expression level in the spike comparing with other four tissues, whereas TaRaf80 exhibited the high abundance in the leaf and TaRaf87 showed high expression levels in root and leaf (Fig. 11a). Under salt stress, TaRaf34 was found to be significantly up-regulated while TaRaf22, TaRaf4 and TaMEKK29 were down-regulated under salt stress condition (Fig. 11b). The qRT-PCR results were highly consistent with that of RNA-seq data, suggesting it is reasonable to use RNA-seq data to assess the expression level of transcripts in wheat and the validated tissues-specific and salt-responsive TaMAKKK provided the candidates for further study of their function in wheat development and stress response.

## Conclusion

This study for the first time identified and characterized the wheat MAPKKK gene family. Through a genome-wide search using the latest available wheat genome information, a total of 155 putative TaMAPKKKs were obtained, which classified into MEKK, ZIK and Raf 3 subfamilies based on the conserved motif signatures. The gene structure, conserved protein domain as well as phylogenetic relationship of these TaMAPKKKs were systematically analyzed and strongly supported the classification. The homologous genes between wheat A, B and D sub-genome and gene duplication were also investigated, which was found to be the main factors contributing to the expansion of wheat MAPKKK gene families. Furthermore, the expression profiles of wheat MAPKKKs during development and under abiotic stresses were investigated and the tissue-specific or stress-responsive TaMAPKKK genes were identified. Finally, 6 tissue-specific and 4 salt-responsive TaMAPKKK genes were selected to validate their expression level through qRT-PCR analysis, which provided the important candidates for further functional analysis of MAPKKK genes in wheat development and stress response. Our current study systematically investigated the genome organization, evolutionary features, regulatory network and expression profiles of the wheat MAPKKK gene family, which not only lay the foundation for investigating the function of these MAPKKKs, but also facilitate to reveal the regulatory and evolutionary mechanism of MAPK cascade involving in growth and development as well as in response to stresses in wheat.

## Additional files

Additional file 1: Accession number and samples information of RNA-seq data using in this study. (XLSX 10 kb) Additional file 2: The primer sequences used for qRT-PCR analysis. (XLSX 8 kb) Additional file 3: Cis-regulatory elements found in the promoters of 155 wheat MAPKKK genes. (XLSX 24 kb) Additional file 4: The chromosome position of the identified homologous TaMAPKKK genes and duplicated genes. (XLSX 12 kb) Additional file 5: The detail of 18 TaMAPKKK orthologous genes in Arabidopsis thaliana. (XLSX 10 kb) Additional file 6: Detail information of the network of TaMAPKKK with other wheat genes. (XLSX 42 kb) Additional file 7: FPKM values of the wheat MAPKKK gene in 5 tissues (grain, root, stem, leaf and spike) and under 4 abiotic stresses (drought, salt, heat and cold). (XLSX 34 kb)
